# Efficacy, Safety, and Role of Antiplatelet Drugs in the Management of Acute Coronary Syndrome: A Comprehensive Review of Literature

**DOI:** 10.7759/cureus.36335

**Published:** 2023-03-18

**Authors:** Muhammad Abubakar, Saud Raza, Khawaja Mushammar Hassan, Izzah Javed, Khawaja Momal Hassan, Fiza Farrukh, Khawaja Muthammir Hassan, Muhammad Ahmad Faraz

**Affiliations:** 1 Department of Internal Medicine, Ameer-ud-Din Medical College, Lahore, PAK; 2 Department of Internal Medicine, Lahore Medical and Dental College, Lahore, PAK; 3 Department of Internal Medicine, Medical University of Silesia, Katowice, POL; 4 Department of Community Medicine, King Edward Medical University, Lahore, PAK; 5 Department of Forensic Medicine, Postgraduate Medical Institute (PGMI), Lahore, PAK

**Keywords:** management, dual antiplatelet therapy, clopidogrel, aspirin, mechanism of action, safety, efficacy, acute coronary syndrome, antiplatelets

## Abstract

Acute coronary syndrome (ACS) is a complex condition characterized by myocardial ischemia or infarction which can lead to significant morbidity and death. Antiplatelet drugs play a crucial role in the management of ACS and have been shown to minimize the incidence of significant adverse cardiovascular events and recurrent myocardial infarction (MI). This comprehensive literature review is intended to summarize current information on the effectiveness, safety, and function of frequently used antiplatelet medications in treating ACS.

Aspirin, clopidogrel, prasugrel, ticagrelor, abciximab, tirofiban, dipyridamole, cilostazol, and novel antiplatelets are included in the review. Aspirin's effectiveness as a first-line antiplatelet medication in ACS is well established. It has significantly lowered the risk of serious adverse cardiovascular events. Clopidogrel, prasugrel, and ticagrelor are P2Y12 receptor inhibitors found to lower the incidence of recurrent ischemia episodes in ACS patients.

Using glycoprotein IIb/IIIa inhibitors such as abciximab, tirofiban, and eptifibatide is effective in managing ACS, especially in high-risk patients. Dipyridamole effectively reduces the risk of recurrent ischemic events in patients with ACS, particularly when used with aspirin. Cilostazol, a phosphodiesterase III inhibitor, has also been shown to reduce the risk of major adverse cardiovascular events (MACE) in patients with ACS.

Antiplatelet drugs' safety in managing ACS has also been well established. Aspirin is generally well-tolerated with a low risk of adverse effects, although the risk of bleeding events, particularly gastrointestinal bleeding, cannot be eliminated. The P2Y12 receptor inhibitors have been associated with a small increase in the risk of bleeding events, particularly in patients with a high risk of bleeding. The glycoprotein IIb/IIIa inhibitors are associated with a higher risk of bleeding than other antiplatelet drugs, especially in high-risk patients.

To summarize, antiplatelet drugs play a crucial role in the management of ACS, and the efficacy and safety of these drugs have been well-established in the literature. The choice of antiplatelet drugs will depend on the patient's risk factors, including age, comorbidities, and bleeding risk. The novel antiplatelets may offer new therapeutic options for managing ACS, and further studies are needed to determine their role in managing this complex condition.

## Introduction and background

Antiplatelet therapy is a well-established treatment option for patients with acute coronary syndrome (ACS). The American Heart Association/American College of Cardiology published a focused update in 2014 on antiplatelet therapy in ACS, which is still widely recognised today [1]. ACS is a term used to describe conditions that include ST-elevation myocardial infarction (STEMI), non-ST elevation myocardial infarction (N-STEMI), and unstable angina. It is a type of coronary heart disease that accounts for one-third of deaths in persons over age 35 [2].

Antiplatelet therapy reduces the risk of thrombosis and its consequences, including death and myocardial infarction (MI). Platelet activation and aggregation in response to endothelial injury is a crucial part of the pathophysiology of ACS, and various antiplatelet therapies target different platelet receptors [3]. Antiplatelet medications are divided into oral and parenteral agents, with oral agents further divided based on the mechanism of action. Aspirin was the first antiplatelet medication and is a cyclooxygenase inhibitor, while other oral antiplatelet medications include clopidogrel, ticagrelor, prasugrel, cilostazol, and dipyridamole [4].

Antiplatelet therapy has evolved over the decades, introducing new agents with increased potency and improving outcomes for patients with ACS. In recent years, potent antiplatelets such as prasugrel and ticagrelor have been added to the treatment regimen, in addition to aspirin, based on the latest guidelines [5]. 

This review provides an overview of the role of antiplatelets in treating ACS. The review will focus on the different types of antiplatelet agents, their mechanisms of action, and their efficacy and safety profiles in managing ACS. The review will also examine the current state of the art, including the latest guidelines, recent trials, and advances in antiplatelet therapy. The review will comprehensively analyse the current evidence and best practices in antiplatelet therapy for ACS. It will provide insights into the future direction of antiplatelet therapy in this field.

## Review

ACS is a group of cardiovascular diseases (CVD) characterized by the sudden onset of chest pain or discomfort. It is caused by the rupture of an atherosclerotic plaque in a coronary artery, leading to the formation of a blood clot that partially or completely occludes the vessel. The formation of the blood clot results from the activation of the coagulation cascade, which is triggered by the exposure of tissue factors from the ruptured plaque to circulating platelets and coagulation factors. Platelets also play a critical role in the development of the clot as they adhere to the site of injury and release their contents, including adenosine diphosphate (ADP) and thromboxane A2 (TXA2). Stent thrombosis is a serious complication that can occur after percutaneous coronary intervention (PCI). The pathophysiology of stent thrombosis is similar to that of ACS, as it also involves the activation of platelets and the coagulation cascade. Stent thrombosis can occur due to multiple factors, including incomplete stent apposition, malposition, stent under-expansion, stent fracture, and patient-related factors such as poor medication adherence and hypercoagulable states.

This literature review will classify antiplatelet agents into six main classes: Irreversible cyclooxygenase inhibitors (aspirin, triflusal), ADP receptor inhibitors (clopidogrel, ticagrelor, ticlopidine, prasugrel, cangrelor), adenosine reuptake inhibitors (dipyridamole), glycoprotein IIb/IIIa platelet inhibitors (abciximab, eptifibatide, tirofiban), protease-activated receptor-1 antagonists (vorapaxar), and phosphodiesterase inhibitors (cilostazol) as shown in Table 1. These antiplatelet agents are used to prevent the formation of blood clots by making platelets less sticky, blocking enzymes involved in clotting, or preventing the activation of platelets.

**Table 1:** Visual Summary of Onset, Mechanism, Duration of action, and Half-life of various Antiplatelet Agents

Antiplatelet agent	Onset	Mechanism	Time to platelet function recovery after drug cessation	Half-life
Aspirin	15-30 min	Irreversible cyclooxygenase inhibitor	8-10 days (platelet lifespan)	15-20 min
Triflusal	1-2 h	Irreversible cyclooxygenase inhibitor	8-10 days (platelet lifespan)	8 h
Clopidogrel	2-4 h	ADP receptor inhibitor (P2Y12)	8-10 days (platelet lifespan)	6 h
Prasugrel	30 min	ADP receptor inhibitor (P2Y12)	8-10 days (platelet lifespan)	8 h
Ticlopidine	24-48 h	ADP receptor inhibitor (P2Y12)	8-10 days (platelet lifespan)	4-5 days
Ticagrelor	30 min	ADP receptor inhibitor (P2Y12)	3-5 days (reversible)	8-12 h
Cangrelor	2-3 min	ADP receptor inhibitor (P2Y12)	30-60 min (reversible)	3-6 min
Abciximab	<2 min	Glycoprotein IIb/IIIa platelet inhibitors	2 days (reversible)	30 min
Eptifibatide	<2 min	Glycoprotein IIb/IIIa platelet inhibitors	4 h (partial recovery)	3 h
Tirofiban	<2 min	Glycoprotein IIb/IIIa platelet inhibitors	4-8 h	2 h
Vorapaxar	60-90 min	Protease-activated receptor-1 antagonist	4-8 weeks	4-12 days
Dipyridamole	24 min	Phosphodiesterase inhibitor	---	11 h

Aspirin, also known as acetylsalicylic acid, is one of the most widely used drugs in the world today. It has been an analgesic, antipyretic, and anti-inflammatory medication in clinical use for over 100 years [6].

ASA, commonly known as aspirin, inhibits the cyclooxygenase (COX) enzyme pathway. Specifically, it is a cyclooxygenase-1 (COX-1) inhibitor that modifies the enzymatic activity of cyclooxygenase-2 (COX-2). Aspirin binding is irreversible, unlike other NSAIDs, such as ibuprofen or naproxen, which bind reversibly to the COX enzyme. COX pathway inhibition redirects arachidonic acids towards the lipoxygenase pathway, generating mostly anti-inflammatory lipoxins. This triggers the production of aspirin-triggered lipoxins, resolvins, and maresins. It also blocks TXA2 on platelets in an irreversible fashion, preventing platelet aggregation. The antiplatelet effect of aspirin makes it a powerful tool in the management of ACS, particularly in the secondary prevention of CVD [7].

Aspirin has also been shown to have three additional modes of action. First, it uncouples oxidative phosphorylation in cartilaginous and hepatic mitochondria by diffusing from the intermembrane space as a proton carrier back into the mitochondrial matrix, where it ionizes once again to release protons. Second, it alters cellular interactions with prostaglandins and leukocyte migration, and third, it induces interferon and activates complement components [7,8].

Several landmark trials have established aspirin's efficacy in managing ACS. In one of the earliest trials, 1266 men with unstable angina were enrolled in a multicenter, double-blind study that showed that aspirin was effective in the primary and secondary prevention of CAD [9]. The results from this and other clinical trials have led to strong guideline recommendations for aspirin use in the acute management and secondary prevention of CVD.

However, aspirin use as a primary prevention strategy is more conservative because recent trials have reported that the benefits of aspirin use in primary prevention are not always clear. The ASPREE and ARRIVE trials compared low-dose aspirin with a placebo among healthy elderly patients. The ASPREE trial found that aspirin did not prevent disability-free survival but did increase major bleeding. Similarly, the ARRIVE trial found that it did not significantly reduce CVD events but did increase gastrointestinal bleeding. The ASCEND trial also found that aspirin reduced CVD events but increased major bleeding [10].

Regarding safety, aspirin has been shown to have several potential toxicities, including neurotoxicity (tinnitus, coma, seizures), renal failure, acute pulmonary edema, and cardiovascular compromise. However, these toxicities are relatively rare and are typically associated with chronic ingestions or high levels of aspirin [11].

In light of the above, aspirin is a powerful and effective tool in managing ACS and the secondary prevention of CVD. The antiplatelet effect of aspirin has been established through several landmark trials and is supported by strong guideline recommendations. However, aspirin use as a primary prevention strategy is more conservative due to concerns about bleeding risk. The best practice in aspirin treatment includes ongoing research and clinical trials to understand better the benefits and risks of aspirin use in various patient populations.

A major advancement in treating patients undergoing PCI has been developing and utilizing agents that block ADP receptors on the platelet membrane, such as thienopyridines [12]. The reason is that ADP is one of the most important mediators of physiologic hemostasis and thrombosis [12]. Intensive oral antiplatelet therapy reduces ischemic events, including stent thrombosis in ACS patients treated with PCI and stenting [13].

The interaction of ADP with its platelet receptors (P2Y1 and P2Y12) plays a crucial role in thrombogenesis. ADP receptor inhibitors are a type of antiplatelet drug used to treat ACS or prevent thromboembolism, MI, or stroke [14]. Thienopyridines are one of the major classes of ADP receptor inhibitors. They prevent platelet aggregation by inhibiting the activation of the IIb/IIIa receptor but do not block the receptor itself. The drugs clopidogrel, prasugrel, ticagrelor, and ticlopidine are classic examples [15].

Clopidogrel is a prodrug often used with aspirin to reduce the risk of subsequent cardiovascular events in ACS patients or who have undergone coronary artery stenting [16]. Studies have shown that clopidogrel, in combination with aspirin, can reduce morbidity and mortality in patients with ACS who are medically managed or undergo PCI [17]. The CAPRIE trial found a statistically significant reduction in the combined incidence of stroke, MI, and vascular death for clopidogrel compared to aspirin, with an 8.7% relative risk reduction [18]. Compared to aspirin, clopidogrel is associated with less severe bleeding and intracranial hemorrhage when taken alone. However, when combined with other antiplatelet agents, the risk of bleeding increases [19]. Approximately 6% of patients experience hypersensitivity to clopidogrel, frequently manifesting as an itchy rash. The symptoms are so intense that the medication has to be discontinued in 1.5% of patients [20]. Clopidogrel resinate, a novel polymeric salt form of clopidogrel, has been evaluated for its efficacy and safety as an anticoagulant drug [21]. 

As a component of dual antiplatelet therapy (DAPT), ticagrelor and prasugrel have better efficacy than clopidogrel in reducing the risk of major adverse cardiovascular events (MACE) but at the cost of a higher bleeding rate [22]. Clopidogrel monotherapy has an advantage in reducing MACE, stroke, and major bleeding events in ACS patients at high risk of bleeding. However, clopidogrel does not work well for everyone because it needs to be activated by an enzyme called CYP450 in the liver. Some people have different versions of this enzyme, making it work faster or slower than normal. These variations are called CYP450 polymorphisms of CYP2C19 variants. The CYP450 polymorphisms can affect how active clopidogrel is in blood and how well it prevents blood clots. People with a slow version of CYP450 may not benefit enough from clopidogrel and may have a higher risk of having another heart attack or stroke. People with a fast version of CYP450 may benefit too much from clopidogrel and may have a higher risk of bleeding [23]. The relative efficacy and safety of ticagrelor and prasugrel in patients with ACS and high bleeding risk (HBR) undergoing PCI remain unclear [24]. A prasugrel dose of 5 mg may cause less bleeding compared with the standard 10 mg dose [25]. The bleeding risk of ticagrelor and prasugrel is not dependent on prior MI status [26]. As ticagrelor is not a prodrug, it has a quicker onset of action and is more potent than clopidogrel. However, it is also associated with similar side effects, such as bleeding and bruising, and is more likely to cause shortness of breath [27]. 

Finally, ticlopidine has an efficacy similar to clopidogrel in preventing stent thrombosis [9]. Nevertheless, it is not used as the first line because of the serious adverse effect of hematologic dyscrasias, with reversible neutropenia being the most common and severe in 0.9% of cases [28]. To summarize, antiplatelet therapy using ADP receptor inhibitors is an important part of medical management for the ACS, with different inhibitors showing varying benefits and limitations. Clopidogrel has shown clinical benefits, but its efficacy is limited by slow onset of action and variability [29]. Both prasugrel and ticagrelor are more effective than clopidogrel in preventing death or adverse cardiac or cerebrovascular events in patients with ACS undergoing PCI. However, the risk of bleeding is unclear [30]. The latest guidelines from ACC recommend careful evaluation of the risks and benefits of pretreatment with P2Y12 receptor inhibitors.

Dipyridamole is a vasodilator and an antiplatelet agent. It functions as a phosphodiesterase inhibitor, raising intracellular levels of cAMP and cGMP by preventing their conversion to AMP and GMP, respectively [31]. Antiplatelet therapy reduces the likelihood of acute coronary thrombosis during routine percutaneous transluminal coronary angioplasty (PTCA) [32]. However, evidence on dipyridamole's efficacy and safety profile specifically for managing ACS is limited. Further studies are needed to assess dipyridamole's effectiveness and safety profile in treating ACS.

Glycoprotein IIb/IIIa inhibitors are a family of medications that reduce platelet aggregation by inhibiting the binding of fibrinogen and von Willebrand factor to glycoprotein IIb/IIIa receptors present on the surface of activated platelets [33]. The glycoprotein IIb/IIIa inhibitors include tirofiban, abciximab, and eptifibatide [34]. These medications have a rapid onset of action, with a plasma half-life of approximately two hours for tirofiban [35]. However, they are only available intravenously and are used as short-term therapy [4]. 

Abciximab, tirofiban, and eptifibatide are antiplatelet drugs used in the treatment of acute myocardial infarction (STEMI) patients undergoing primary PCI [36]. They are typically used in the usual care, including aspirin, clopidogrel, prasugrel, and heparin or bivalirudin. Abciximab and tirofiban are usually given as an IV bolus 10 to 60 minutes before the commencement of PCI, followed by a continuous infusion. Studies have compared the efficacy of tirofiban, abciximab, and eptifibatide, but the results have been inconclusive. However, one study found that tirofiban was more effective than the standard of care for STEMI and non-STEMI acute coronary syndrome (NSTE ACS) patients undergoing planned PCI and receiving medical management [37,38]. Another study found that tirofiban and abciximab were equally effective [39]. 

Abciximab was studied in three phase-III clinical trials (EPIC, EPILOG, and CAPTURE) and is a prescription-only medication indicated for intravenous use. However, it is no longer manufactured, and further studies are necessary to determine its efficacy [38,39]. Tirofiban and eptifibatide are low-molecular-weight competitive and reversible GPIIb/IIIa antagonists that act on the α IIb-subunit of GPIIb/IIIa. Tirofiban can be used in place of abciximab with a bolus dose followed by a continuous infusion [40]. Tirofiban is associated with a lower risk of thrombocytopenia than abciximab [41]. The American College of Chest Physicians (ACCP) suggests monitoring the activated partial thromboplastin time (aPTT) or activated clotting time (ACT) before removing the arterial sheath when administering tirofiban and only removing the sheath if the aPTT is less than 45 seconds or the ACT is less than 180 seconds [42].

In a nutshell, both abciximab and tirofiban have unique administration and efficacy profiles. Further studies are needed to fully understand their efficacy and safety profiles in the management of ACS.

Eptifibatide is an antiplatelet drug used to manage ACS. It works by inhibiting glycoprotein IIb/IIIa receptors found in platelets. Eptifibatide has been indicated by the Food and Drug Administration (FDA) for use in ACS through studies like PURSUIT and IMPACT-II [41]. However, It can induce profound thrombocytopenia, a relatively rare side effect that can lead to significant patient morbidity [41]. It is important to monitor blood counts and observe patients while they are on eptifibatide treatment. The efficacy and safety of eptifibatide in patients with ACS are still unclear and a potential research area [43].

Cangrelor is a P2Y12 receptor antagonist used during the PCI to reduce the risk of MI, repeat coronary revascularization, and stent thrombosis [44]. It is used as an adjunctive treatment with aspirin in patients with ACS undergoing PCI. Clinical trials have shown that it reduces ischemic events when combined with clopidogrel [45]. Cangrelor has a rapid onset of action (2-3 minutes) and recovery of platelet function after discontinuation, with a plasma half-life of 3-6 minutes [46,47]. It is not cleared by the kidneys and does not require dose adjustment in patients with renal failure [47]. A cangrelor-clopidogrel combination is relatively safe and more effective than clopidogrel alone in urgent and elective PCI settings. The benefits of the combination are evident when using the universal definition of MI [48].

Cangrelor is the only intravenous P2Y12 receptor inhibitor currently available, with a rapid onset and offset of action compared to oral P2Y12 inhibitors like clopidogrel, prasugrel, and ticagrelor [49]. A study found that it can be used as a safe alternative to lumbar drains, with the drains able to be removed 2.5 hours after the infusion [50]. A study comparing cangrelor and eptifibatide discovered that both drugs have comparable safety and efficacy as a bridge treatment for patients with recent coronary stents. Additionally, substituting cangrelor with eptifibatide in select patients could result in cost savings [51].

Cilostazol is a medication commonly used in antiplatelet therapy after PCI in patients with ACS. The long-term effect of cilostazol therapy has yet to be completely discovered, but several studies have reported the potential benefits and side effects [4]. Cilostazol is a phosphodiesterase III inhibitor and an antiplatelet agent. It reversibly inhibits platelet aggregation and has vasodilatory and antiproliferative properties [52]. Cilostazol also inhibits adenosine reuptake and the upregulation of adenylate cyclase activity, resulting in increased cAMP levels [53]. 

There are some studies suggesting cilostazol's efficacy in reducing recurrent ischemic events. However, more data is needed [54]. One study found that prolonged dual-antiplatelet therapy (DAPT), including cilostazol, might decrease the risk of mortality, MACE, and stroke in patients with chronic kidney disease (CKD) without any significant difference in bleeding or revascularization [55].

On the other hand, cilostazol is contraindicated in patients with heart failure of any severity as it is an inhibitor of phosphodiesterase III and has caused decreased survival compared to placebo in patients with class III-IV heart failure [52]. Common side effects of cilostazol therapy include headache (34%), diarrhea (19%), palpitations (10%), and a slight elevation in heart rate (5 to 7 beats/min) [52]. However, cilostazol may also cause serious side effects, such as chest pain, pounding heartbeats, light-headedness, agranulocytosis, mouth sores, easy bruising, unusual bleeding, and purple or red spots under the skin [56]. Overall, cilostazol's safety and efficacy in managing ACS require further investigation.

Vorapaxar is a novel antiplatelet drug that acts as a protease-activated receptor-1 (PAR-1) antagonist. It works by inhibiting thrombin-related platelet aggregation, an action different compared to other antiplatelet medications such as aspirin and P2Y12 inhibitors [57]. The efficacy of vorapaxar in managing ACS has been evaluated in several studies, but the results are mixed. The result of one trial found that vorapaxar did not significantly reduce the primary end point of death from cardiovascular causes, MI, stroke, recurrent ischemia with rehospitalization, or urgent coronary revascularization. However, vorapaxar significantly reduced the secondary end point of death from cardiovascular causes, MI, or stroke. Vorapaxar also significantly increased the risk of moderate or severe bleeding, especially in patients with a history of stroke [58]. Similarly, in the Thrombin Receptor Antagonist for Clinical Event Reduction in Acute Coronary Syndrome (TRACER) trial, the drug revealed new safety data but the trial's primary endpoint in an ACS population still needs to be met [59]. More studies are needed to establish its safety as part of DAPT before it is welcomed in clinical practice. Whether or not to use it as a monotherapy is still unclear.

Triflusal is an antiplatelet drug that is structurally related to aspirin. It acts on different targets involved in platelet aggregation and vascular inflammatory processes and increases nitric oxide synthesis in neutrophils, resulting in increased vasodilatory potential [60]. It is considered a preferential COX-2 inhibitor antiplatelet agent and is as effective as aspirin in preventing serious vascular events [61]. Studies have been conducted to evaluate the safety and efficacy of triflusal in patients with aspirin hypersensitivity and ACS. These studies showed that triflusal had good tolerability in these patients and was associated with a low rate of adverse events and bleeding events [62]. One study evaluated the safety and efficacy of triflusal in patients with ACS and aspirin hypersensitivity, and the results showed that triflusal was well tolerated in these patients. The same study showed that in patients with aspirin hypersensitivity treated with a coronary stent, triflusal use was associated with a low rate of adverse events in the long term. No cases of stent thrombosis or hypersensitivity reactions to triflusal were reported [63]. Another study was conducted to assess the tolerability of triflusal in patients with aspirin-exacerbated respiratory disease (AERD), and the results showed that triflusal was well tolerated in these patients [61]. More studies are needed to establish the role of triflusal as a safe and effective alternative for patients with ACS and other similar conditions.

DAPT is the gold standard for the prevention of thrombotic complications in ACS patients and those undergoing PCI. DAPT consists of a combination of aspirin and a P2Y12 inhibitor, which works by inhibiting platelets and preventing the formation of clots in the blood vessels. According to the recent trials, DAPT is recommended for a minimum of 12 months after drug-eluting stent (DES) PCI, but the optimal duration is unclear. Some trials have suggested that shorter durations of DAPT (3 or 6 months) may be associated with lower bleeding risk and similar ischemic risk compared to longer durations (12 or 30 months), especially in patients with HBR. However, these trials have some limitations, such as heterogeneity of patient populations, stent types, DAPT regimens, and outcome definitions. Therefore, it is important to individualize DAPT duration based on patient characteristics, clinical presentation, stent type, bleeding risk, and ischemic benefit [64,65].

The primary efficacy outcome of DAPT is to minimize the risk of MACE such as cardiovascular death, MI, and stroke [66]. The primary safety outcome is minimizing major or minor bleeding risk. The safety profile of DAPT is favorable, with low bleeding risk, but there is a trade-off with a higher risk of other bleeding events [67].

## Conclusions

ACS is a serious condition that requires prompt medical attention. The current state of the art in treating ACS involves using antiplatelet drugs, specifically P2Y12 receptor inhibitors, which prevent the aggregation of platelets and reduce the risk of thrombosis. The latest guidelines for managing ACS recommend using ADP receptor inhibitors, such as clopidogrel, ticagrelor, and prasugrel, combined with aspirin as early as possible after the onset of symptoms. The current state of the art in aspirin treatment includes ongoing research and clinical trials to understand better the benefits and risks of aspirin use in various patient populations. Advances in ADP receptor inhibitor therapy have led to the development of more potent and faster-acting drugs, such as ticagrelor and prasugrel, showing varying benefits and limitations in reducing the risk of MACE. Overall, the use of ADP receptor inhibitors in combination with therapies other than aspirin continues to be an important aspect of managing ACS. Ongoing research is aimed at improving the efficacy and safety of these drugs.

Both abciximab and tirofiban have their unique administration and efficacy profiles. The safety and efficacy of eptifibatide in patients with ACS are still unclear. More research is needed to completely comprehend the effectiveness and safety profiles of eptifibatide, cangrelor, cilostazol, vorapaxar, and triflusal in treating ACS.
